# Causal effects of atrial fibrillation on brain white and gray matter volume: a Mendelian randomization study

**DOI:** 10.1186/s12916-021-02152-9

**Published:** 2021-11-24

**Authors:** Sehoon Park, Soojin Lee, Yaerim Kim, Semin Cho, Kwangsoo Kim, Yong Chul Kim, Seung Seok Han, Hajeong Lee, Jung Pyo Lee, Soryoung Lee, Eue-Keun Choi, Kwon Wook Joo, Chun Soo Lim, Yon Su Kim, Dong Ki Kim

**Affiliations:** 1 Department of Biomedical Sciences, Seoul National University College of Medicine, Seoul, Korea; 2 Department of Internal Medicine, Armed Forces Capital Hospital, Gyeonggi-do, Seongnam, Korea; 3 Department of Internal Medicine, Department of Internal Medicine, Uijeongbu Eulji University Medical Center, Gyeonggi-do, Uijeongbu, Korea; 4 Department of Internal Medicine, Seoul National University Hospital, 101 Daehak-ro, Jongno-gu, Seoul, 03080 Korea; 5 Department of Internal Medicine, Seoul National University College of Medicine, Seoul, Korea; 6 Department of Internal Medicine, Keimyung University School of Medicine, Daegu, Korea; 7 Transdisciplinary Department of Medicine & Advanced Technology, Seoul National University Hospital, Seoul, Korea; 8 Kidney Research Institute, Seoul National University, Seoul, Korea; 9 Department of Internal Medicine, Seoul National University Boramae Medical Center, Seoul, Korea

**Keywords:** Atrial fibrillation, Brain, Stroke, Mendelian randomization

## Abstract

**Background:**

Atrial fibrillation (AF) and brain volume loss are prevalent in older individuals. We aimed to assess the causal effect of atrial fibrillation on brain volume phenotypes by Mendelian randomization (MR) analysis.

**Methods:**

The genetic instrument for AF was constructed from a previous genome-wide association study (GWAS) meta-analysis (15,993 AF patients and 113,719 controls of European ancestry). The outcome summary statistics for head-size-normalized white or gray matter volume measured by magnetic resonance imaging were provided by a previous GWAS of 33,224 white British participants in the UK Biobank. Two-sample MR by the inverse variance–weighted method was performed, supported by pleiotropy-robust MR sensitivity analysis. The causal estimates for the effect of AF on ischemic stroke were also investigated in a dataset that included the findings from the MEGASTROKE study (34,217 stroke patients and 406,111 controls of European ancestry). The direct effects of AF on brain volume phenotypes adjusted for the mediating effect of ischemic stroke were studied by multivariable MR.

**Results:**

A higher genetic predisposition for AF was significantly associated with lower grey matter volume [beta −0.040, standard error (SE) 0.017, *P*=0.017], supported by pleiotropy-robust MR sensitivity analysis. Significant causal estimates were identified for the effect of AF on ischemic stroke (beta 0.188, SE 0.026, *P*=1.03E−12). The total effect of AF on lower brain grey matter volume was attenuated by adjusting for the effect of ischemic stroke (direct effects, beta −0.022, SE 0.033, *P*=0.528), suggesting that ischemic stroke is a mediator of the identified causal pathway. The causal estimates were nonsignificant for effects on brain white matter volume as an outcome.

**Conclusions:**

This study identified that genetic predisposition for AF is significantly associated with lower gray matter volume but not white matter volume. The results indicated that the identified total effect of AF on gray matter volume may be mediated by ischemic stroke.

**Supplementary Information:**

The online version contains supplementary material available at 10.1186/s12916-021-02152-9.

## Background

Atrial fibrillation (AF) is the most common cardiac arrhythmia associated with the risk of stroke, heart failure, dementia, and mortality [1] and further contributes to a substantial socioeconomic burden [2]. The prevalence of AF is substantially increasing along with the global aging trend [3, 4].

As AF is highly prevalent in elderly individuals, cognitive dysfunction or functional brain disorders, which are also common in elderly people, have been associated with AF [5]. In addition, brain volume loss is related to persistent AF, along with low cerebral blood flow in AF patients [6–8]. However, demonstration of the causal effect of AF on structural brain volume changes has yet to be performed. Because pathologic brain volume loss and AF share risk factors and are both common in older individuals with multiple comorbidities, whether the observed low brain volume is a consequence of AF could hardly be answered by observational studies due to residual confounding effects. In addition, whether AF alone can cause brain volume loss even without stroke needs to be studied, as previous reports assessed the association between AF and dementia in stroke-free individuals, and brain volume loss was present before the identified first stroke event in AF patients [5, 9, 10]. Such evidence for the causal effect of AF on brain volume and its mechanistic association with stroke would suggest whether accelerated brain volume loss in AF patients may be ameliorated through appropriate AF management targeting the risk of ischemic stroke.

Mendelian randomization (MR) is an analytic method that can identify causal estimates with epidemiologic data [11]. MR utilizes a genetic instrument that is fixed before birth; thus, instrumented genetic predisposition is minimally affected by confounders or reverse causation. The significant association between genetic predisposition, which would result in a higher occurrence of the exposure of interest, and the outcome would suggest the presence of a causal effect of the exposure. MR has been widely introduced in the medical literature and has identified an important causal linkage between complex exposures and outcomes [12].

In this study, we performed a summary-level MR analysis to demonstrate the causal effects of AF on brain volume phenotypes. We also performed an MR analysis to investigate the causal pathway between AF, stroke, and brain white or gray matter volumes. We hypothesized that AF would decrease a certain type of brain volume that would be potentially mediated by ischemic stroke.

## Methods

### Study setting

**Fig. 1:**
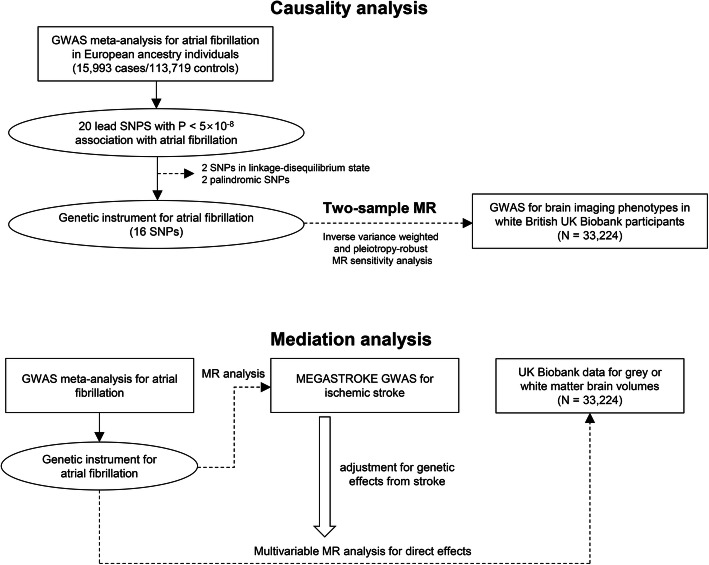
Flow diagram of the study

The study was a summary-level MR analysis that mainly consisted of two parts (Fig. 1). The causal estimates for the effect of AF, genetically predicted by single-nucleotide polymorphisms (SNPs) reported in a previous genome-wide association study (GWAS), on white or gray matter volume, measured in the independent UK Biobank data, were initially tested by two-sample MR. Next, additional GWAS results for ischemic stroke phenotype were utilized, and the causal effects of AF on ischemic stroke awere investigated. Finally, to determine whether the effects of AF on brain volume are mediated by ischemic stroke, multivariable MR analysis adjusted for the genetic effects of ischemic stroke was performed.

### Genetic instrument for AF

Data from a previous GWAS meta-analysis for AF were used for this study [13, 14]. Unlike from our previous study also investigating the causal estimates from AF [12, 15], as the outcome data solely consisted of the UK Biobank cohort, we intentionally used the previous GWAS meta-analysis, which did not include the UK Biobank data, to maintain the two-sample design [13]. Two-sample MR has strength in a conservative sense over an MR analysis with overlapping samples, as the potential bias from weak instruments is toward false-negative results; thus, a positive finding from two-sample MR can be more robust evidence for the presence of causality [16]. In addition, considering the outcome sample size, applying a MR design including sample overlap was considered to cause substantial bias towards confounding effects [17] and a bias by pleiotropic effect was suspected when we used the most recent GWAS meta-analysis data as the genetic instruments for AF (Additional File 1: Supplemental Methods and Additional File 1: Supplemental Table 1).

As the outcome summary statistics for brain volume were limited to those with white British ancestry, we downloaded the summary statistics for AF in individuals of European ancestry, including 15,993 AF cases and 113,719 controls. Within the European ancestry-specific analysis, 20 SNPs in different genetic loci were reported to have a genome-wide significant (*P* < 5×10^-8^) association with the AF trait (Additional File 1: Supplemental Table 2). To ensure the independence of the instruments, we disregarded rs10800507 and rs35176054 as having weaker associations with AF but being in a linkage disequilibrium state with other variants, leaving 18 independent SNPs (within a 1 Mb window, *r*^2^ < 0.001). As strand alignment is uncertain for palindromic SNPs, we additionally disregarded 2 palindromic SNPs (rs2921421 and rs651386) in the main genetic instruments for AF. We also performed a sensitivity analysis preserving the two palindromic SNPs or disregarding only rs651386, which had intermediate allele frequency, as the genetic instruments for AF. We inspected the orientation of the genetic effects and secured that the direction was from exposure towards outcome variable by using Steiger filtering [18].

### MR assumptions

MR analysis requires three key assumptions to be attained by the genetic instrument to demonstrate causal effects [11]. First, the relevance assumption is that the genetic instrument should be strongly associated with the exposure of interest. A previous GWAS meta-analysis already provided SNPs with strong association strength with AF. We additionally tested the association strength in the UK Biobank data in 337,138 unrelated individuals of white British ancestry who passed the sample quality control filter by calculating the explained variance for AF by polygenic score analysis [12]. The AF data were collected from the hospital admission records or main causes of death, identified by an International Classification of Diseases (ICD)-10 diagnostic code of I48 or ICD-9 diagnostic code of 4273 [12]. The explained variance for AF by polygenic score derived by the instrumented SNPs was calculated by McFadden’s pseudo-*R* square method. The *F* statistic, which should be over 10 to avoid weak instrument bias [19], was calculated to confirm the attainment of the relevance assumption, by the below equation: [(n-k-1)/(k)]*[*R*^2^/(1-*R*^2^)], where *n* represents sample size, *k* represents number of instruments, and *R*^2^ represents explained variance of the exposure phenotype [19]. Furthermore, we additionally validated whether the genetically predicted AF by the current instruments relevantly predicts kidney function impairment as the positive control outcome. The CKDGen GWAS meta-analysis for chronic kidney disease traits (41,395 cases and 439,303 controls, URL: https://ckdgen.imbi.uni-freiburg.de/) was assessed as the outcome data by the summary-level MR method described below [20, 21]. Chronic kidney disease was reported to be causally affected by AF in our previous MR analysis including two independent cohorts but with different genetic instruments for AF [12, 21].

Second, the independence assumption is that the genetic instrument should not be associated with confounders. To attain this assumption, we investigated whether a SNP had a strong (*P* < 1×10^-5^) association with hypertension, obesity, diabetes mellitus, dyslipidemia, and thyroid disorder in the abovementioned 337,138 white British UK Biobank participants by performing a GWAS adjusted for age, sex, age×sex, age^2^, and the first 10 principal components (Additional File 1: Supplemental Table 2). Furthermore, in summary-level MR, we tested the presence of directional pleiotropy by identifying MR-Egger intercepts [22]. In addition, we performed multiple pleiotropy-robust MR sensitivity analysis to derive the causal estimates with relaxation of the assumption [23].

Third, the exclusion restriction assumption is that the causal effect of interest should be through the studied exposure. Although a formal test for this assumption is not yet possible, the utilized median-based method can relax this assumption for up to 50% of the instrumented weights, providing sensitivity analysis for the attainment of this assumption [24].

### Outcome data for brain volume traits

A recent GWAS for brain imaging traits in the UK Biobank was performed for 33,224 white British ancestry individuals aged 40 to 69 years [25]. The summary statistics for white and gray matter volumes were downloaded and used as the outcome data (URL: https://open.win.ox.ac.uk/ukbiobank/big40/) [26]. The study identified that brain imaging phenotypes are mostly genetically trackable and reported functionally relevant genetic information associated with brain structures. The brain volume was measured by magnetic resonance imaging, and the study provided summary statistics for quantile-normalized brain volume phenotypes and for the phenotypes adjusted for head size. Among the brain volume phenotypes measured, we aimed to assess the causal estimates toward composite brain gray or white matter volume, which has been repetitively investigated in previous observational studies [6, 7]. As total gray or white matter volume would generally reflect the sum of variance, this approach would secure the statistical power of an MR analysis, which is particularly important in two-sample MR, which may be biased toward false-negative findings.

### Summary-level MR analysis methods

The main MR method was the multiplicative random-effect inverse variance–weighted method, which allows balanced pleiotropy [27].

As unbalanced pleiotropic effects may still bias the causal estimates by the inverse variance–weighted method, additional pleiotropy-robust MR sensitivity analyses are commonly performed [16, 23]. First, MR-Egger regression with bootstrapped standard error was performed with the test for the presence of directional pleiotropy or MR-Egger intercept [22]. The method has strength in that the presence of a directional pleiotropic effect can be statistically tested and that pleiotropy-robust causal estimates can be yielded. However, MR-Egger regression has weak statistical power, particularly when the number of instrumented SNPs is low, and can still be biased when the untestable Instrument Strength Independent of Direct Effect assumption is violated by a group of variants acting through the same pleiotropic pathway [28]. Thus, additional sensitivity analysis by the median-based method is recommended; thus, we performed the weighted median method, which allows up to 50% invalid instrumented weights [24]. Next, the MR pleiotropy residual sum and outlier test, which detects and corrects the effects of outliers, was performed when the test for global heterogeneity was significant [29]. We also performed MR–robust adjusted profile score, which provides robust causal estimates by modeling the pleiotropic effects, assuming the effects are normally distributed [30]. The summary-level MR analysis was performed by the TwoSampleMR package in *R* (version 4.0.2, the *R* foundation) [31].

As we tested two outcome phenotypes, gray and white matter volumes, a two-sided *P* value < 0.05/2 by the inverse variance–weighted method indicated significance in the main causality analysis for the causal estimates of AF on brain matter volume phenotypes. As other sensitivity analyses were performed to support the main results, we used the conventional significance threshold (*P* < 0.05) to characterize a sensitivity analysis result as supportive of the main result. However, as MR-Egger regression has weak statistical power, the causal estimates by the MR-Egger method were interpreted by general effect sizes and whether a significant directional pleiotropic effect was identified by the MR-Egger intercept *P* value [23, 32].

Post hoc power calculation for MR analysis was performed by an online tool (URL: https://sb452.shinyapps.io/power/), followed the method suggested by S. Burgess (URL: http://mendelianrandomization.com/) [33, 34]. We calculated beta^2*2*MAF*(1-MAF), where MAF indicates minor allele frequency, of each instrumented SNP and summed the values for the coefficient necessary for the power calculator. As information for prevalence ratio was unavailable, although beta should be scaled to a prevalence increase of a unit of an exposure, we used the odds ratios as the proximate of the prevalence ratio considering that the case proportion was not high (12.6%) in the GWAS meta-analysis data which was used to generate genetic instruments. The point causal estimate towards normalized brain gray or white matter volume by the inverse variance–weighted method was used as the effect size of the causal effect.

### Analysis with ischemic stroke as a mediator

Multivariable MR analysis with direct adjustment for the effects of other phenotypes of the utilized genetic instrument has been used to investigate whether the identified total effect in univariable MR is mediated by a potential phenotype [35–37]. Considering that AF is well known to be associated with ischemic stroke, we performed additional mediation analysis including the data from a GWAS meta-analysis (34,217 ischemic stroke cases 406,111 controls) from individuals of European ancestry by the MEGASTROKE consortium (URL: https://www.megastroke.org/) [38, 39].

To establish the causal pathways, we first tested the causal estimates of AF on ischemic stroke by the aforementioned summary-level MR methods. Finally, we calculated the direct effect of AF, predicted by the main instruments including 16 SNPs, on brain volume phenotypes by multivariable MR adjusted for the genetic effects of ischemic stroke (Additional File 1: Supplemental Table 2). Indirect effects were not calculated because linear relation between the tested variables, which is required to calculate indirect effects, was not secured as binary exposure was being tested [40]. The multivariable analysis was performed by inverse-variance-weighted method and MR-Egger regression, using the MVMR and MendelianRandomization package in R [41, 42]. Again, the Bonferroni-corrected significance level (*P* < 0.05/2) was used to indicate significant findings in the main causal estimates, and the conventional threshold (*P* < 0.05) was used in other sensitivity analyses.

The causal estimates from ischemic stroke on brain volume phenotypes were not calculated because the available genome-wide significant 32 SNPs identified by the MEGASTROKE study explained limited variance of ischemic stroke phenotype, leading to a *F* statistic of 9.4 indicating that a valid two-sample MR with sufficient power was impossible with the available data.

## Results

### Genetic instrument for AF

None of the lead SNPs associated with AF with genome-wide significance showed a strong association with a potential confounder in the UK Biobank data (Additional File 1: Supplemental Table 2). Among the 337138 individuals of white British ancestry of the UK Biobank, the median age was 58 years old, and 46.3% were males. From this group, we identified 15,446 (4.6%) AF cases. The polygenic score calculated by the genetic instrument was strongly associated (*P* < 2×10^-16^), with phenotypical AF explaining 1.6% of the variance; thus, the *F* statistic of the 16 SNPs in the outcome data of 33,224 individuals was 33.7. When we applied genetic instruments to the positive control outcome, kidney function impairment, genetically predicted AF was relevantly associated with a higher risk of chronic kidney disease (Additional File 1: Supplemental Table 3).

### Causal estimates of AF on brain volume

**Fig. 2:**
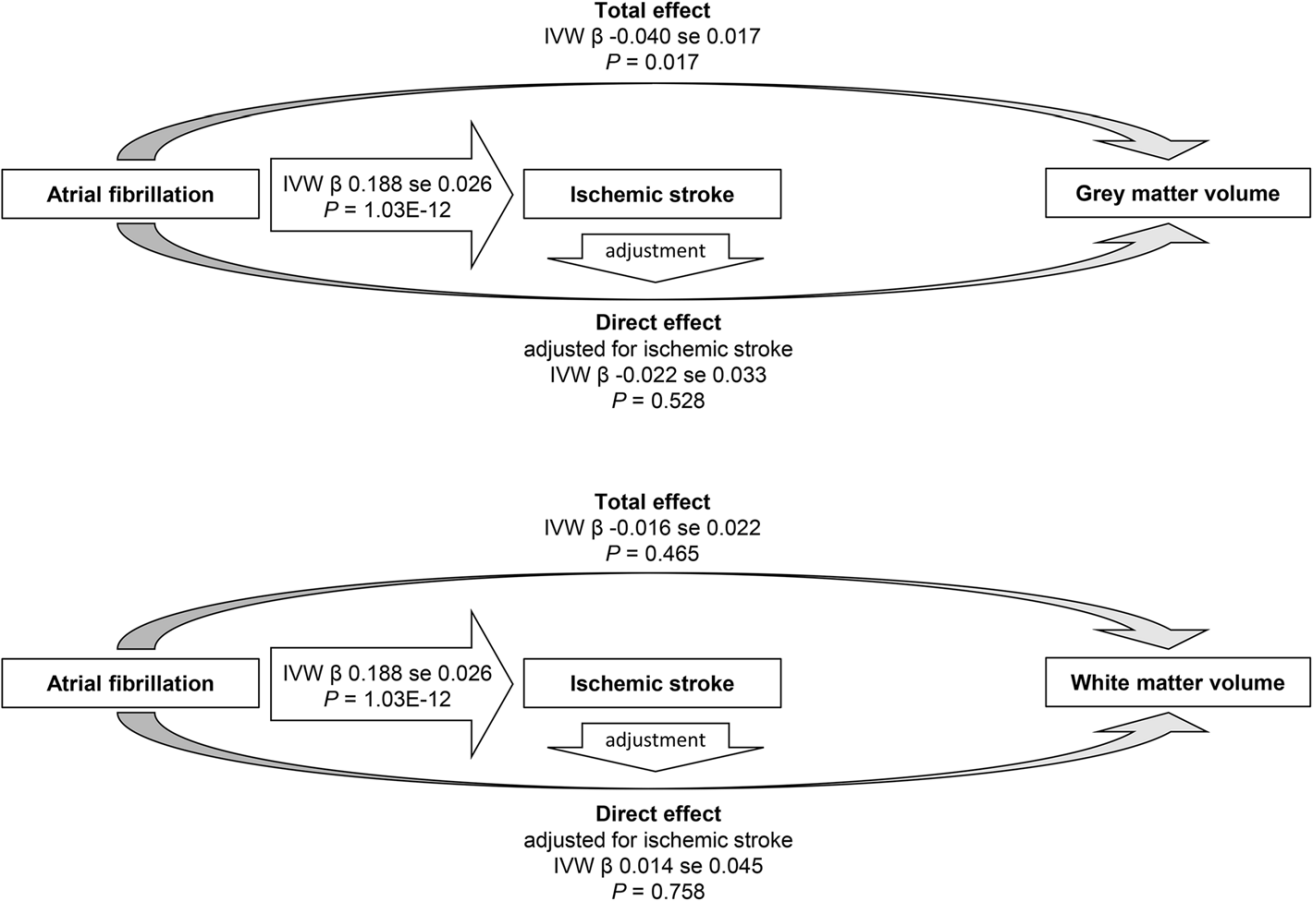
Schematic drawing for the mediation analysis. The causal estimates determined by the inverse variance–weighted (IVW) method are presented. The causal estimates toward brain volume phenotypes are from the analysis assessing head-size-normalized outcomes. The total effects were the causal estimates from two-sample MR with genetically predicted atrial fibrillation as exposure and brain volume phenotypes as outcomes. The direct effects were the causal estimates from the two-sample MR, which were adjusted for the genetic effects from ischemic stroke by multivariable MR analysis. MR Mendelian randomization

**Fig. 3:**
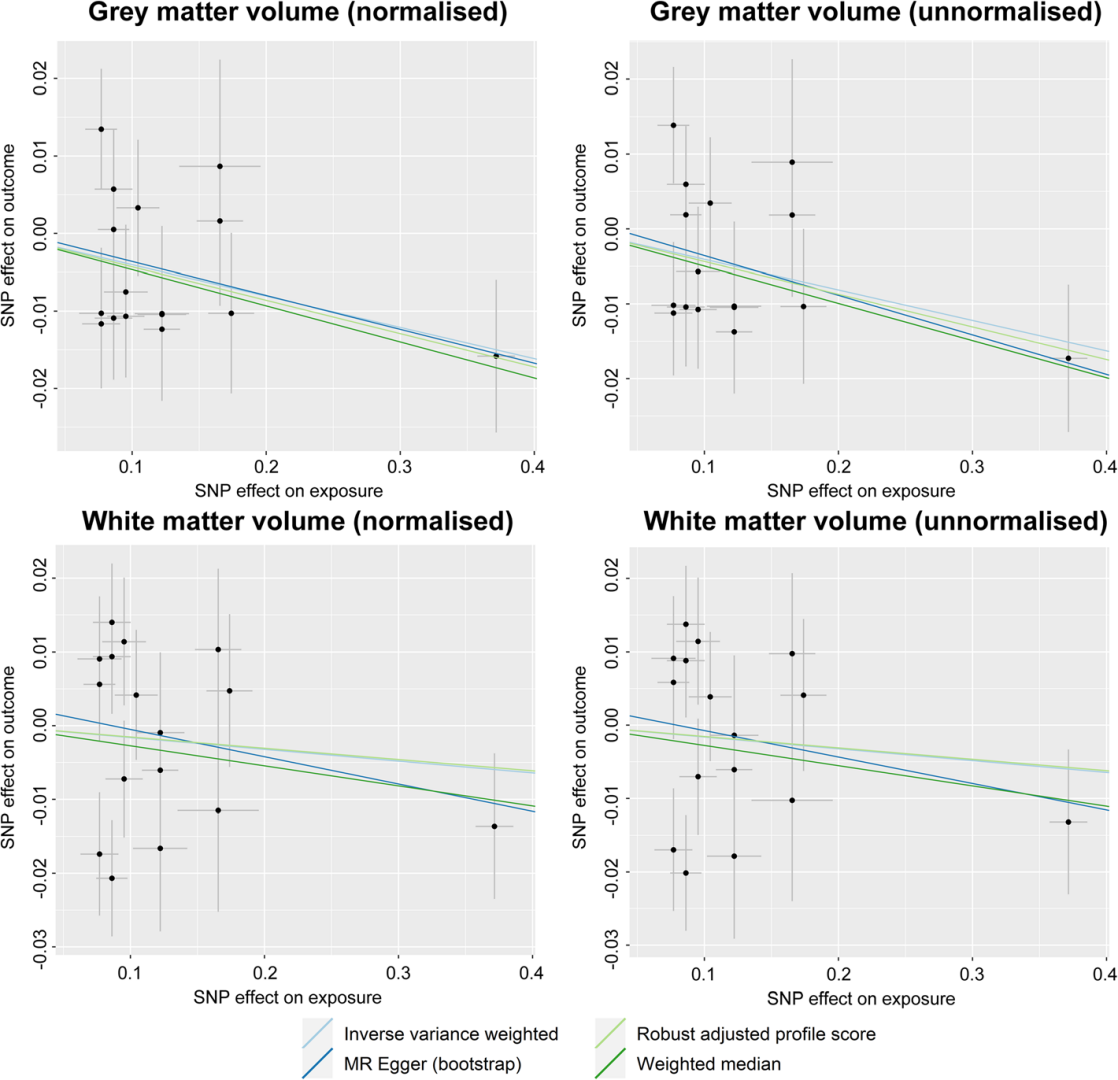
Scatter plot of the Mendelian randomization analysis showing the causal estimates of atrial fibrillation on brain volume phenotypes

The summary-level MR results indicated that genetic predisposition for AF was significantly associated with lower gray matter volume, both normalized and unnormalized (Figs. 2 and 3 and Table 1). The causal estimates were also significant by all performed MR sensitivity analysis methods, except for the MR-Egger regression analysis. However, the MR-Egger intercept *P* value indicated the absence of a directional pleiotropic effect, and the effect size of the causal estimates by MR-Egger regression was generally similar to the other methods. On the other hand, the causal estimates for white matter volume did not show a significant association between genetic predisposition for AF and normalized or unnormalized white matter volume by MR analysis.

**Table 1:** Causal estimates of atrial fibrillation on brain gray or white matter volume in the UK Biobank participants by summary-level Mendelian randomization

Genetically predicted exposure	Outcome phenotype	MR-Egger intercept *P* value	Cochran’s *Q* statistic *P* value for heterogeneity	MR method	Beta	Standard error	*P* value
Atrial fibrillation (16 SNPs)	Gray matter volume (normalized)	0.984	0.547	IVW	−0.040	0.017	0.017
				MR-Egger (bootstrap)	−0.044	0.040	0.147
				Weighted median	−0.047	0.023	0.044
				MR-RAPS	−0.043	0.017	0.014
				MR-PRESSO	NA	NA	NA
	Gray matter volume (unnormalized)	0.817	0.499	IVW	−0.041	0.017	0.015
				MR-Egger (bootstrap)	−0.052	0.040	0.089
				Weighted median	−0.050	0.023	0.033
				MR-RAPS	−0.044	0.017	0.013
				MR-PRESSO	NA	NA	NA
	White matter volume (normalized)	0.549	0.041	IVW	−0.016	0.022	0.465
				MR-Egger (bootstrap)	−0.038	0.042	0.186
				Weighted median	−0.027	0.023	0.242
				MR-RAPS	−0.015	0.022	0.483
				MR-PRESSO	NA	NA	NA
	White matter volume (unnormalized)	0.553	0.053	IVW	−0.016	0.022	0.464
				MR-Egger (bootstrap)	−0.038	0.041	0.181
				Weighted median	−0.028	0.024	0.241
				MR-RAPS	−0.016	0.022	0.470
				MR-PRESSO	NA	NA	NA

*MR* Mendelian randomization, *IVW* inverse variance–weighted, *RAPS* robust adjusted profile score, *PRESSO* pleiotropy residual sum and outlier, *SNP* single-nucleotide polymorphism

MR-PRESSO analysis was performed, but the MR-PRESSO global test for heterogeneity did not identify correctable effects of outliers

The units of the causal estimates were log odds ratio for the cause (atrial fibrillation) and standard deviation for the effect (brain volume)

When we additionally included 2 palindromic SNPs or inferred strand information to genetically predict AF, similar findings were replicated, as genetically predicted AF was significantly associated with lower brain gray matter volume (Additional File 1: Supplemental Table 4 and Additional File 1: Supplemental Table 5). The MR-Egger regression again yielded marginal findings with generally comparable effect sizes. Otherwise, the causal estimates toward brain white matter volume remained nonsignificant.

The power towards brain gray matter volume of the summary-level MR analysis was 79.1% when the 16 SNPs and was 87.3% when 18 SNPs, including palindromic ones, were used to genetically predict AF. However, the power was weak towards brain white matter volume, as it was 23.6% by 18 SNPs and 19.7% by 16 SNPs, respectively.

### AF, ischemic stroke, and brain volume

The causal estimates of AF on ischemic stroke indicated a significant causal effect, and the causal estimates were significant by all performed MR analysis methods (Table 2 and Fig. 2). Although a directional pleiotropic effect was suspected by the MR-Egger intercept *P* value (directional pleiotropy *P* = 0.028), MR-Egger regression correcting the potential pleiotropic effect still provided significant causal estimates.

**Table 2:** Causal estimates of atrial fibrillation on ischemic stroke

Genetically predicted exposure	Outcome phenotype	MR-Egger intercept *P* value	Cochran’s Q statistic *P* value for heterogeneity	MR method	Beta	Standard error	*P* value
Atrial fibrillation (16 SNPs)	Ischemic stroke	0.028	0.068	IVW	0.188	0.026	1.03E−12
				MR-Egger (bootstrap)	0.251	0.047	< 0.001
				Weighted median	0.240	0.030	4.83E−16
				MR-RAPS	0.201	0.025	5.57E−16
				MR-PRESSO	NA	NA	NA

*MR* Mendelian randomization, *IVW* inverse variance–weighted, *RAPS* robust adjusted profile score, *PRESSO* pleiotropy residual sum and outlier, *SNP* single-nucleotide polymorphism

MR-PRESSO analysis was performed, but as the MR-PRESSO global test for heterogeneity did not identify correctable effects of outliers, the causal estimates were the same as those of the inverse variance–weighted method

The units of the causal estimates were log odds ratio for the cause (atrial fibrillation) and log odds ratio for the effect (stroke)

Finally, when the abovementioned total effects from AF on brain volume phenotypes were adjusted for the genetic effects of ischemic stroke by multivariable MR, the causal estimates on brain gray matter volume were attenuated to the point of nonsignificance (Table 3 and Fig. 2). Namely, the direct effects of AF on brain gray matter volume were nonsignificant independent of the effects of ischemic stroke. The causal estimates by the multivariable MR analysis for white matter volume remained nonsignificant as the total effects. However, the overall conditioned *F* statistics indicated the possibility of weak instruments in the multivariable MR anlaysis, suggesting that it was difficult to independently predict the atrial fibrillation and ischemic stroke phenotypes.

**Table 3:** Direct effects of atrial fibrillation on brain volume phenotypes adjusted for genetic effects of ischemic stroke by multivariable MR analysis.

Genetically predicted exposure	Conditional *F* statistics (atrial fibrillation)	Conditional *F* statistics (stroke)	Outcome phenotype	Heterogeneity *Q* statistics *P* value	Method	Beta	Standard error	*P* value
Atrial fibrillation, adjusted for genetic effects of ischemic stroke (16 SNPs)	1.734	1.406	Gray matter volume (normalized)	0.443	IVW	−0.022	0.033	0.528
					MR-Egger	−0.001	0.065	0.992
			Gray matter volume (unnormalized)	0.397	IVW	−0.022	0.034	0.533
					MR-Egger	−0.015	0.066	0.823
			White matter volume (normalized)	0.035	IVW	0.014	0.045	0.758
					MR-Egger	−0.003	0.088	0.971
			White matter volume (unnormalized)	0.043	IVW	0.013	0.045	0.777
					MR-Egger	−0.005	0.087	0.958

*MR* Mendelian randomization, *IVW* inverse variance-weighted, *SNP* single-nucleotide polymorphism

Multivariable inverse variance-weighted method and MR-Egger regression was performed to yield the causal estimates

## Discussion

This study identified that genetic predisposition for AF is significantly associated with lower gray matter volume but not white matter volume. With our efforts to attain the MR assumptions, our study supports that AF is a causative factor for lower gray matter volume. The results further suggest causal pathways from AF to low gray matter volume may be mediated by ischemic stroke.

An observational association between AF and functional brain disorders, representatively dementia, has been reported [5, 9]. Historically, loss in brain gray matter volume and increase in abnormal white matter volume were considered to be associated with dementia severity [43]. As AF was associated with lower gray matter volume by observational findings [6, 7], structural brain atrophy caused by AF was suspected to be one of the mechanisms of cognitive impairment in AF patients. In addition, as AF is a widely recognized risk factor for ischemic stroke, either by cardioembolism or not [44], and ischemic stroke has been reported to be associated with accelerated brain atrophy [45], conceptional linkage between AF, ischemic stroke, and brain volume loss has been suggested. On the other hand, there have been debates regarding whether AF may be associated with brain volume loss or the risk of dementia independent of stroke-related mechanisms [7, 9]. However, confirming the causal linkage between AF, ischemic stroke, and brain volume loss was difficult by traditional study designs, as observational findings are prone to residual confounding effects or reverse causation [11]. Moreover, as brain volume is a relatively unique phenotype available in a large number of individuals and rarely sequentially measured, an observational study investigating the causal effect of AF on brain volume is difficult to perform. In this study, we implemented MR analysis to test causal estimates from exposure and predicted complex outcomes using the genetic instrument. Finally, we identified that AF may be causally linked to a lower gray matter volume. The identified causal estimates were dependent on the effect of ischemic stroke; thus, the study suggests causal pathways linking AF, ischemic stroke, and lower brain gray matter volume.

Based on the study results, as AF is a causative factor for lower brain gray matter volume, appropriate AF management might delay dementia-related brain atrophy. Considering that the identified causal pathway may be mediated by ischemic stroke, conventional clinical interventions lowering the risk of ischemic stroke in AF patients may be beneficial for preventing accelerated loss of gray matter volume. Recent studies reported that AF patients who received a rhythm-control strategy exhibited a lower risk of cognitive function impairment, supporting that the identified causal pathway may be extended toward cognitive function outcome [46, 47]. A future study would be needed to test our hypothesis that appropriate anticoagulative therapy or rhythm-control intervention in patients with AF would decrease the risk of brain volume loss in patients with AF. In addition, considering that subclinical AF and early brain disorders are often underdiagnosed, AF screening in the high-risk elderly population with brain atrophy or cognitive dysfunction would be considered to facilitate early diagnosis of the suggested causal factor.

In this study, the multivariable MR analysis indicated that it was difficult to independently predict the two closely tight phenotypes, AF and ischemic stroke, by genetic information considering the low conditioned *F* statistics. However, if we allow the interpretation for the results considering the effects from AF on brain volume is hardly explained apart from the close bound between AF and ischemic stroke, suggests that the stroke-mediated pathway would be the prioritized biological linkage rather than a stroke-independent effect of AF on low brain gray matter volume. Previous observational findings suggest that AF was associated with dementia or brain volume independent of stroke might have been potentially affected by confounding effects, as AF commonly occurs in individuals with multiple underlying diseases [8, 9]. However, caution is necessary for such interpretation, as the modest degree of direct effect of AF on brain volume phenotypes, not mediated by ischemic stroke, could have been unrevealed because of the potential false-negative bias in two-sample MR [16], particularly considering the low conditioned *F* statistics of AF phenotype when adjusted for the genetic effects towards ischemic stroke trait. As AF is linked to diverse biological consequences, including subclinical brain hemodynamic compromises or neurohormonal responses [8, 48, 49], a future study is still necessary to investigate whether ischemic stroke–independent effects of AF on brain volume or cognitive function are present.

There are several limitations to this study. First, the study assumes that the brain volumes of middle to elderly UK Biobank participants were determined by degenerative processes later in life. Although this assumption may be attained because brain volume changes are largely affected by risk factors for degenerative diseases [50], it is still possible that developmental, rather than degenerative, determinants might have affected the brain volume phenotypes. To overcome this issue, we also investigated the brain volume phenotypes adjusted for head size, which may partially reflect some developmental differences. However, it should be noted that attainment of the exclusion-restriction assumption, which is untestable, is required to consider our MR analysis results valid. Second, we assessed binary AF exposure in this MR analysis; however, AF has diverse severity and status. Thus, whether the effects of AF are different according to the subtypes (e.g., paroxysmal or persistent) could not be studied herein. Third, the study is mainly based on data from individuals of European ancestry; thus, generalizability to other ethnic populations is not guaranteed. MR results can also be affected by selection bias; thus, future replication studies may be necessary to confirm that the findings could be applied to the general population. Fourth, as the effect size of the causal estimates from AF towards brain white matter volume was modest than that towards brain gray matter volume, statistical power was weak for the phenotype. As two-sample MR can be biased towards false-negative finding, a modest degree of causal effect from AF towards brain white matter volume cannot be disregarded from this study. Lastly, the multivariable MR analysis used instruments with low conditioned *F* statistics. Also, the causal estimates from stroke phenotypes on brain volume were not calculated because of the limited availability of instruments. This limitation of the available dataset should be overcome in future studies to clearly dissect the linkage between AF, ischemic stroke, and brain volumes.

## Conclusions

In conclusion, our MR results suggested that AF was causally linked to a lower brain gray matter volume, possibly mediated by the effects of ischemic stroke. A future study is warranted to investigate whether appropriate AF management may result in delayed progression of gray matter volume loss in AF patients.

## Supplementary Information

**Additional File 1: Supplemental Methods and Supplemental Table 1-Supplemental Table 5.** Supplemental Methods – The reasons that another, more recent GWAS for atrial fibrillation was not selected for the dataset implemented to develop genetic instruments. **Supplemental Table 1.** Results of the MR analysis including overlapping samples using the 84 SNPs from the most recent GWAS meta-analysis as the genetic instruments for atrial fibrillation. **Supplemental Table 2.** Genetic instrument for atrial fibrillation developed from the GWAS within the individuals of European ancestry. **Supplemental Table 3** – Causal estimates from atrial filtration on chronic kidney disease as positive outcome in the CKDGen GWAS data. **Supplemental Table 4.** Causal estimates from atrial fibrillation on brain grey or white matter volume of the UK Biobank participants by summary-level Mendelian randomization including palindromic SNPs. **Supplemental Table 5.** Causal estimates from atrial fibrillation on brain grey or white matter volume of the UK Biobank participants by summary-level Mendelian randomization inferring strand information

## Data Availability

The data for this study are available in the public domain, as described in the manuscript.

## Abbreviations

AF: Atrial fibrillation
GWAS: Genome-wide association study
ICD: International classification of diseases
MAF: Minor allele frequency
MR: Mendelian randomization
SNP: Single-nucleotide polymorphism
