# Supplementary material for: Causal effects of atrial fibrillation on brain white and gray matter volume: a Mendelian randomization study
Source: BMC Med. 2021 Nov 24;19:274. doi: 10.1186/s12916-021-02152-9 (PMC8611907; doi:10.1186/s12916-021-02152-9)
Supplement: Supplementary file 1 — Additional File 1: Supplemental Methods and Supplemental Table 1-Supplemental Table 5. Supplemental Methods – The reasons that another, more recent GWAS for atrial fibrillation was not selected for the dataset implemented to develop genetic instruments. Supplemental Table 1. Results of the MR analysis including overlapping samples using the 84 SNPs from the most recent GWAS meta-analysis as the genetic instruments for atrial fibrillation. Supplemental Table 2. Genetic instrument for atrial fibrillation developed from the GWAS within the individuals of European ancestry. Supplemental Table 3 – Causal estimates from atrial filtration on chronic kidney disease as positive outcome in the CKDGen GWAS data. Supplemental Table 4. Causal estimates from atrial fibrillation on brain grey or white matter volume of the UK Biobank participants by summary-level Mendelian randomization including palindromic SNPs. Supplemental Table 5. Causal estimates from atrial fibrillation on brain grey or white matter volume of the UK Biobank participants by summary-level Mendelian randomization inferring strand information [file 12916_2021_2152_MOESM1_ESM.pdf]

Online Supplemental Material

Causal effect of atrial fibrillation on brain white or grey matter volume: Mendelian randomization study

Park et al.

Supplemental Methods. The reasons that another, more recent GWAS for atrial fibrillation was not selected for the dataset implemented to develop genetic instruments.

Supplemental Table 1. Results of the MR analysis including overlapping samples using the 84 SNPs from the most recent GWAS meta-analysis as the genetic instruments for atrial fibrillation.

Supplemental Table 2. Genetic instrument for atrial fibrillation developed from the GWAS within the individuals of European ancestry.

Supplemental Table 3. Causal estimates from atrial filtration on chronic kidney disease as positive outcome in the CKDGen GWAS data.

Supplemental Table 4. Causal estimates from atrial fibrillation on brain grey or white matter volume of the UK Biobank participants by summary-level Mendelian randomization including palindromic SNPs.

Supplemental Table 5. Causal estimates from atrial fibrillation on brain grey or white matter volume of the UK Biobank participants by summary-level Mendelian randomization inferring strand information.

Supplemental Methods. The reasons that another, more recent GWAS for atrial fibrillation was not selected for the dataset implemented to develop genetic instruments.

The most recent GWAS for atrial fibrillation by Roseli C et al. included larger sample size and reported a larger number of SNPs associated with atrial fibrillation phenotype [1]. However, we did not use the data to develop genetic instruments for this study, because of the below critical issues.

First, the GWAS meta-analysis included the UK Biobank data, which was the outcome dataset for brain volume phenotypes in the current MR analysis. This means that the outcome data completely overlapped with the dataset used to develop genetic instruments, similarly as a one-sample MR analysis which is different from the current two-sample MR analysis. It is well known that such MR analysis with overlapping samples can be biased towards observational findings. In addition, the previous literature identified that such MR analysis would be invalid for an outcome dataset with a sample size lower than 100,000 individuals [2]. Considering that the current outcome brain volume phenotypes were measured in 33,244 individuals, allowing the complete overlap would lead to critical bias.

Second, increasing the number of genetic variants to explain a phenotype is not always helpful to increase instrumental power. Considering the calculation of F statistics, including SNPs that are not contributing a large portion to the variance explained would even decrease the instrumental power [3]. Therefore, including a larger number of SNPs but also some weak variants would decrease the instrumental power, which causes a critical bias towards observational findings particularly for an MR analysis including overlapping samples.

Third, increasing wider ranges of SNPs has some weakness if weakly associated variants are included, as pleiotropic SNPs that are tightly bound to confounding phenotypes but weakly to the exposure phenotype of interest may be present. Different from our main MR analysis, if we use the 84 SNPs from the largest and most recent GWAS (results from the European ancestry-specific analysis) as the genetic instruments for atrial fibrillation [1], the MR-Egger regression intercept P value, which is a former test to suspect a pleiotropic effect, indicated that some directional pleiotropic effect was present.

Fourth, the result made us to suspect that the overall issues led to a bias in the causal estimates, as the causal estimates from the MR-Egger regression, correcting such pleiotropic effect, and other MR analysis were different. The pleiotropy-robust MR analysis results indicated the significant causal effect from atrial fibrillation on brain volume phenotypes, however, the main causal estimates by the inverse-variance weighted method were null, which further raised suspicion for the bias by pleiotropic effect or issues related to weak instruments (Supplemental Table 1). Also, the weighted median method provided marginal findings, thus, for conclusive results, we required the different instruments with clear-cut results.

Therefore, we used the previous GWAS to secure the two-sample MR analysis setting, which is more conservative because even a potential bias from weak instruments is present the direction would be towards a false-negative result. Namely, a positive finding from a two-sample MR is more likely to reflect the presence of a true causal effect. In addition, the instrumental power was secured, a potential directional pleiotropic effect was not present for the main findings considering the MR-Egger intercept P values, and the main MR analysis and pleiotropy-robust methods provided consistent findings.

[1] Roselli C, Chaffin MD, Weng LC, Aeschbacher S, Ahlberg G, Albert CM, Almgren P, Alonso A, Anderson CD, Aragam KG et al: Multi-ethnic genome-wide association study for atrial fibrillation. Nat Genet 2018, 50(9):1225-1233.
[2] Minelli C, Del Greco MF, van der Plaat DA, et al. The use of two-sample methods for Mendelian randomization analyses on single large datasets. Int J Epidemiol 2021 doi: 10.1093/ije/dyab084 [published Online First: 2021/04/27]
[3] Burgess S, Thompson SG; CRP CHD Genetics Collaboration. Avoiding bias from weak instruments in Mendelian randomization studies. Int J Epidemiol. 2011;40(3):755-764. doi:10.1093/ije/dyr036

Supplemental Table 1. Results of the MR analysis including overlapping samples using the 84 SNPs from the most recent GWAS meta-analysis as the genetic instruments for atrial fibrillation.

Genetically predicted exposure	Outcome phenotype	MR-Egger intercept P value	Cochran's statistics Q P value for hegerogeneity	MR method	beta	standard error	P value
Atrial fibrillation (84 SNPs)	Grey matter volume (normalised)	0.230	< 0.001	IVW	-0.013	0.017	0.446
				MR-Egger (bootstrap)	-0.068	0.025	0.006
				Weighted median	-0.040	0.021	0.059
				MR-RAPS	-0.019	0.017	0.256
				MR-PRESSO	-0.025	0.014	0.081
	Grey matter volume (unnormalised)	0.204	< 0.001	IVW	-0.013	0.017	0.423
				MR-Egger (bootstrap)	-0.070	0.025	0.001
				Weighted median	-0.043	0.022	0.053
				MR-RAPS	-0.019	0.017	0.254
				MR-PRESSO	-0.026	0.014	0.073
	White matter volume (normalised)	0.025	< 0.001	IVW	-0.004	0.016	0.776
				MR-Egger (bootstrap)	-0.042	0.024	0.042
				Weighted median	-0.035	0.022	0.115
				MR-RAPS	-0.013	0.015	0.387
				MR-PRESSO	-0.016	0.013	0.251
	White matter volume (unnormalised)	0.024	< 0.001	IVW	-0.004	0.016	0.793
				MR-Egger (bootstrap)	-0.041	0.025	0.046
				Weighted median	-0.034	0.021	0.110
				MR-RAPS	-0.013	0.015	0.402
				MR-PRESSO	-0.012	0.014	0.396

MR = Mendelian randomization, IVW = Inverse variance weighted, RAPS = Robust Adjusted Profile Score, PRESSO = Pleiotropy ReSidual Sum and Outlier

The unit of causal estimates were from a Z-score unit (atrial fibrillation) towards a standard deviation (brain volume).

Supplemental Table 2. Genetic instrument for atrial fibrillation developed from the GWAS within the individuals of European ancestry.

Bais information			Variant filter		Exposure association			Potential confounder association											
								Diabetes mellitus			Dyslipidemia			Hypertension			Obesity		
RSID	effect allele	other allele	Linkage disequilibrium	Palindromic	beta	SE	P	OR	SE	P	OR	SE	P	OR	SE	P	OR	SE	P
rs10800507	C	G	TRUE, rs651386	TRUE	0.086	0.014	1.87E-11	0.997	0.012	8.17E-01	1.008	0.007	2.19E-01	1.006	0.006	3.58E-01	0.999	0.006	8.42E-01
rs10824026	A	G			0.122	0.018	8.29E-11	1.024	0.016	1.39E-01	0.999	0.010	8.76E-01	0.979	0.009	1.42E-02	1.019	0.008	1.80E-02
rs11264280	T	C			0.122	0.014	2.77E-17	0.983	0.012	1.63E-01	0.997	0.007	6.37E-01	0.994	0.007	4.06E-01	1.017	0.006	5.49E-03
rs11598047	G	A			0.166	0.017	3.16E-21	0.993	0.016	6.58E-01	0.999	0.010	8.97E-01	1.004	0.009	6.66E-01	0.979	0.008	9.21E-03
rs11773845	A	C			0.095	0.014	3.35E-13	0.987	0.012	2.79E-01	1.008	0.007	2.78E-01	0.995	0.006	4.34E-01	0.992	0.006	1.57E-01
rs12664873	T	G			0.077	0.016	1.80E-08	1.001	0.013	9.19E-01	1.006	0.007	4.34E-01	0.993	0.007	2.73E-01	1.003	0.006	6.63E-01
rs2106261	T	C			0.174	0.017	4.01E-24	0.981	0.015	2.07E-01	1.001	0.009	9.20E-01	0.982	0.008	2.74E-02	0.988	0.008	1.27E-01
rs2129977	A	G			0.372	0.014	7.25E-136	0.968	0.015	2.89E-02	1.007	0.009	3.89E-01	1.014	0.008	8.71E-02	1.008	0.007	2.57E-01
rs2723064	T	C			0.086	0.014	1.88E-10	1.042	0.012	5.29E-04	1.014	0.007	4.51E-02	1.025	0.006	1.24E-04	1.008	0.006	2.03E-01
rs281868	G	A			0.077	0.012	1.03E-08	0.975	0.011	3.06E-02	0.997	0.007	6.10E-01	1.012	0.006	6.52E-02	0.991	0.006	1.26E-01
rs2921421	G	C		TRUE	0.542	0.099	3.29E-08	1.113	0.168	5.26E-01	1.124	0.101	2.48E-01	1.020	0.096	8.36E-01	0.945	0.088	5.19E-01
rs35176054	A	T	TRUE, rs11598047	TRUE	0.131	0.018	1.75E-11	0.995	0.018	7.63E-01	0.996	0.010	6.83E-01	1.009	0.010	3.40E-01	0.990	0.009	2.51E-01
rs62133983	G	T			0.086	0.014	1.36E-10	1.008	0.012	4.88E-01	1.003	0.007	6.12E-01	0.994	0.006	3.66E-01	0.994	0.006	2.75E-01
rs651386	A	T		TRUE	0.104	0.014	6.23E-15	1.010	0.012	3.95E-01	1.003	0.007	7.09E-01	1.004	0.006	4.83E-01	0.997	0.006	5.61E-01
rs6864727	C	T			0.077	0.014	1.12E-08	0.988	0.012	3.47E-01	1.000	0.007	9.68E-01	1.003	0.007	7.11E-01	1.007	0.006	2.77E-01
rs7026071	T	C			0.086	0.012	2.86E-11	0.999	0.012	9.18E-01	1.007	0.007	3.03E-01	1.005	0.006	4.44E-01	1.001	0.006	8.68E-01
rs7183206	A	G			0.122	0.020	7.70E-12	0.970	0.017	6.77E-02	0.985	0.010	1.29E-01	0.974	0.009	3.85E-03	0.983	0.008	3.54E-02
rs7508	A	G			0.095	0.016	6.34E-10	1.009	0.013	5.00E-01	1.017	0.008	2.30E-02	0.996	0.007	5.24E-01	0.996	0.006	5.37E-01
rs75190942	A	C			0.166	0.030	2.82E-08	0.988	0.020	5.50E-01	0.998	0.012	8.58E-01	0.979	0.011	4.93E-02	1.011	0.010	2.54E-01
rs883079	T	C			0.104	0.016	1.31E-13	1.006	0.013	6.67E-01	1.016	0.008	4.15E-02	0.999	0.007	8.65E-01	0.999	0.006	8.76E-01

	Potential confounder association				Outcome association												Ischemic stroke association		
	Thyroid disease			Lowest P with	Grey matter volume (normalised)			Grey matter volume (unnormalised)			White matter volume (normalised)			White matter volume (unnormalised					
RSID	OR	SE	P	a confounder	beta	SE	P	beta	SE	P	beta	SE	P	beta	SE	P	beta	SE	P
rs10800507	1.006	0.011	5.89E-01	2.19E-01	0.010	0.008	2.06E-01	0.010	0.008	2.14E-01	-0.009	0.008	2.69E-01	-0.008	0.008	2.82E-01	0.0142	0.0102	0.1656
rs10824026	1.007	0.016	6.79E-01	1.42E-02	-0.010	0.011	3.39E-01	-0.010	0.011	3.37E-01	-0.001	0.011	8.99E-01	-0.001	0.011	9.31E-01	0.0055	0.0141	0.6951
rs11264280	1.017	0.012	1.74E-01	5.49E-03	-0.012	0.008	1.35E-01	-0.014	0.008	9.66E-02	-0.006	0.008	4.63E-01	-0.006	0.008	4.64E-01	-0.0012	0.0109	0.9148
rs11598047	1.002	0.016	9.03E-01	9.21E-03	0.002	0.011	8.83E-01	0.002	0.011	8.65E-01	0.010	0.011	3.71E-01	0.010	0.011	3.43E-01	0.018	0.0135	0.1826
rs11773845	0.995	0.012	6.60E-01	1.57E-01	-0.011	0.008	1.78E-01	-0.011	0.008	1.74E-01	-0.007	0.008	3.74E-01	-0.007	0.008	3.61E-01	0.0133	0.0101	0.1897
rs12664873	0.992	0.012	5.13E-01	2.73E-01	-0.010	0.008	2.22E-01	-0.010	0.008	2.27E-01	0.009	0.008	2.79E-01	0.009	0.008	2.82E-01	0.0071	0.0111	0.5242
rs2106261	1.023	0.015	1.39E-01	2.74E-02	-0.010	0.010	3.20E-01	-0.010	0.010	3.17E-01	0.004	0.010	6.93E-01	0.005	0.010	6.48E-01	0.0493	0.013	0.000157
rs2129977	1.027	0.014	6.55E-02	2.89E-02	-0.016	0.010	1.09E-01	-0.017	0.010	7.97E-02	-0.013	0.010	1.81E-01	-0.014	0.010	1.67E-01	0.091	0.0119	2.65E-14
rs2723064	1.003	0.012	8.04E-01	1.24E-04	-0.011	0.008	1.72E-01	-0.010	0.008	1.92E-01	0.014	0.008	8.54E-02	0.014	0.008	7.96E-02	0.0212	0.01	0.03445
rs281868	1.000	0.011	9.84E-01	3.06E-02	0.013	0.008	8.29E-02	0.014	0.008	7.45E-02	0.006	0.008	4.51E-01	0.006	0.008	4.69E-01	0.0014	0.01	0.8871
rs2921421	1.092	0.169	6.02E-01	2.48E-01	0.145	0.120	2.27E-01	0.152	0.120	2.06E-01	0.103	0.120	3.92E-01	0.110	0.120	3.60E-01	NA	NA	NA
rs35176054	0.996	0.018	8.33E-01	2.51E-01	0.015	0.012	2.06E-01	0.016	0.012	1.84E-01	0.007	0.012	5.34E-01	0.007	0.012	5.58E-01	0.0173	0.014	0.2167
rs62133983	1.005	0.011	6.52E-01	2.75E-01	0.006	0.008	4.62E-01	0.006	0.008	4.43E-01	0.009	0.008	2.57E-01	0.009	0.008	2.28E-01	-0.0114	0.0099	0.2508
rs651386	1.018	0.012	1.14E-01	1.14E-01	-0.005	0.008	5.26E-01	-0.005	0.008	5.14E-01	-0.006	0.008	4.74E-01	-0.006	0.008	4.60E-01	0.0163	0.01	0.1037
rs6864727	1.029	0.012	1.83E-02	1.83E-02	-0.012	0.008	1.63E-01	-0.011	0.008	1.78E-01	-0.017	0.008	4.19E-02	-0.017	0.008	3.71E-02	0.0153	0.0107	0.152
rs7026071	0.996	0.012	7.32E-01	3.03E-01	0.001	0.008	9.49E-01	0.002	0.008	8.11E-01	-0.020	0.008	1.08E-02	-0.021	0.008	8.85E-03	0.0138	0.0101	0.1701
rs7183206	0.991	0.017	5.82E-01	3.85E-03	-0.010	0.011	3.60E-01	-0.010	0.011	3.61E-01	-0.018	0.011	1.14E-01	-0.017	0.011	1.41E-01	0.0287	0.0146	0.0498
rs7508	1.029	0.013	2.82E-02	2.30E-02	-0.008	0.009	3.84E-01	-0.006	0.009	5.10E-01	0.011	0.009	1.86E-01	0.011	0.009	1.88E-01	0.0183	0.0113	0.1055
rs75190942	0.991	0.020	6.54E-01	4.93E-02	0.009	0.014	5.28E-01	0.009	0.014	5.16E-01	-0.010	0.014	4.55E-01	-0.012	0.014	4.02E-01	0.0427	0.02	0.03246
rs883079	1.005	0.013	6.81E-01	4.15E-02	0.003	0.009	7.08E-01	0.003	0.009	6.96E-01	0.004	0.009	6.60E-01	0.004	0.009	6.37E-01	0.0291	0.0111	0.008929

OR = odds ratio, SE = standard ratio

Linkage disequilibrium was identified by applying threshold of r2 < 0.001 within 1 Mb-window.

Supplemental Table 3. Causal estimates from atrial filtration on chronic kidney disease as positive outcome in the CKDGen GWAS data.

Genetically predicted exposure	Outcome phenotype	MR-Egger intercept P value	Cochran's statistics Q P value for hegerogeneity	MR method	beta	standard error	P value
Atrial fibrillation (16 SNPs)	Chronic kidney diasese	0.731	0.033	IVW	0.066	0.026	0.012
				MR-Egger (bootstrap)	0.107	0.043	0.004
				Weighted median	0.080	0.027	0.003
				MR-RAPS	0.071	0.029	0.013
				MR-PRESSO	NA	NA	NA

MR = Mendelian randomization, IVW = Inverse variance weighted, RAPS = Robust Adjusted Profile Score, PRESSO = Pleiotropy ReSidual Sum and Outlier

MR-PRESSO analysis was performed but as MR-PRESSO global test for heterogeneity did not identify correctable effects from outliers.

The unit of causal estimates were from a log odds ratio (atrial fibrillation) towards a log odds ratio (chronic kidney disease) or log-estimated glomerular filtration rate.

Supplemental Table 4. Causal estimates from atrial fibrillation on brain grey or white matter volume of the UK Biobank participants by summary-level Mendelian randomization including palindromic SNPs.

Genetically predicted exposure	Outcome phenotype	MR-Egger intercept P value	Cochran's statistics Q P value for hegerogeneity	MR method	beta	standard error	P value
Atrial fibrillation (18 SNPs)	Grey matter volume (normalised)	0.898	0.549	IVW	-0.039	0.016	0.017
				MR-Egger (bootstrap)	-0.045	0.031	0.075
				Weighted median	-0.044	0.022	0.047
				MR-RAPS	-0.042	0.017	0.014
				MR-PRESSO	NA	NA	NA
	Grey matter volume (unnormalised)	0.942	0.492	IVW	-0.039	0.016	0.016
				MR-Egger (bootstrap)	-0.051	0.032	0.047
				Weighted median	-0.047	0.023	0.042
				MR-RAPS	-0.042	0.017	0.013
				MR-PRESSO	NA	NA	NA
	White matter volume (normalised)	0.639	0.059	IVW	-0.017	0.021	0.416
				MR-Egger (bootstrap)	-0.035	0.033	0.159
				Weighted median	-0.030	0.023	0.197
				MR-RAPS	-0.015	0.021	0.472
				MR-PRESSO	NA	NA	NA
	White matter volume (unnormalised)	0.639	0.076	IVW	-0.017	0.020	0.404
				MR-Egger (bootstrap)	-0.032	0.032	0.173
				Weighted median	-0.029	0.023	0.203
				MR-RAPS	-0.015	0.021	0.456
				MR-PRESSO	NA	NA	NA

MR = Mendelian randomization, IVW = Inverse variance weighted, RAPS = Robust Adjusted Profile Score, PRESSO = Pleiotropy ReSidual Sum and Outlier

MR-PRESSO analysis was performed, but the MR-PRESSO global test for heterogeneity did not identify correctable effects of outliers.

The unit of causal estimates were from a log odds ratio (atrial fibrillation) towards a standard deviation (brain volume).

Supplemental Table 5. Causal estimates from atrial fibrillation on brain grey or white matter volume of the UK Biobank participants by summary-level Mendelian randomization inferring strand information.

Genetically predicted exposure	Outcome phenotype	MR-Egger intercept P value	Cochran's statistics Q P value for hegerogeneity	MR method	beta	standard error	P value
Atrial fibrillation (17 SNPs)	Grey matter volume (normalised)	0.915	0.478	IVW	-0.039	0.017	0.019
				MR-Egger (bootstrap)	-0.043	0.032	0.097
				Weighted median	-0.046	0.023	0.043
				MR-RAPS	-0.041	0.017	0.018
				MR-PRESSO	NA	NA	NA
	Grey matter volume (unnormalised)	0.925	0.423	IVW	-0.039	0.017	0.021
				MR-Egger (bootstrap)	-0.050	0.032	0.045
				Weighted median	-0.050	0.022	0.027
				MR-RAPS	-0.042	0.017	0.016
				MR-PRESSO	NA	NA	NA
	White matter volume (normalised)	0.592	0.045	IVW	-0.015	0.022	0.493
				MR-Egger (bootstrap)	-0.032	0.033	0.173
				Weighted median	-0.027	0.023	0.246
				MR-RAPS	-0.013	0.022	0.544
				MR-PRESSO	NA	NA	NA
	White matter volume (unnormalised)	0.594	0.059	IVW	-0.015	0.021	0.479
				MR-Egger (bootstrap)	-0.031	0.033	0.173
				Weighted median	-0.027	0.024	0.246
				MR-RAPS	-0.014	0.021	0.525
				MR-PRESSO	NA	NA	NA

MR = Mendelian randomization, IVW = Inverse variance weighted, RAPS = Robust Adjusted Profile Score, PRESSO = Pleiotropy ReSidual Sum and Outlier

MR-PRESSO analysis was performed, but the MR-PRESSO global test for heterogeneity did not identify correctable effects of outliers.

The unit of causal estimates were from a log odds ratio (atrial fibrillation) towards a standard deviation (brain volume).
